# Assessment of the Effects of Seed Storage Time on Germination Rate and Performance Evaluation of Ethiopian Faba Bean (*Vicia faba* L.) Varieties for Yield and Related Traits

**DOI:** 10.1155/2022/6338939

**Published:** 2022-05-05

**Authors:** Fekadu Gadissa, Zemedkun Kassaye, Solomon Abiyu

**Affiliations:** Madda Walabu University, POB–247, Bale Robe, Ethiopia

## Abstract

In Ethiopia, faba bean (*Vicia faba* L.) varieties are important and widely used as a source of food and cash source to large number of subsistence farmers. However, their production and productivity is below the world's average partly because of lack of their sustainable performance in the current scenario of climate change. Therefore, the present study was designed to test the effects of seed storage time and to evaluate the performance of 31 faba bean varieties collected from Holeta, Kulumsa and Sinana agricultural research systems, Ethiopia. The study involved germination test and field experiment that was laid out using randomized complete block design (RCBD) and conducted at multiple test locations. Data were collected on qualitative and quantitative traits and analyzed using SAS version 9.0, and MINITAB® Release 19. Accordingly, most of the varieties showed a promising germination rate regardless of their storage duration suggesting their sustainable performance under suitable storage conditions. Most of the qualitative and quantitative traits showed a wide range of variations revealing their stable performance and better chance for further improvement. Analysis of variance also revealed a highly significant (*p* < 0.001) variation for several of the traits suggesting maintenance of the original diversity that could be important in further selection breeding. Likewise, high genetic advance coupled with high heritability and genotypic coefficient of variation together with wide range of variations in both PCV and GCV observed in several of the quantitative traits suggest their sustainable performance and significance in further effective selection. Moreover, a promising high yielding varieties such as Dida1, Welki, Hachalu, Ashebeka and Obse have been identified for further use. Clustering grouped the varieties into three clusters implying significant amount of genetic variability among them. Overall, the results generated could be used as a baseline information for improving faba bean production and productivity. However, to exploit more and determine the actual performance of the varieties more markers such as molecular markers (DNA based) are recommended.

## 1. Introduction

In Ethiopian agricultural system, pulses are one of the most valuable crops to smallholder farmers. They serve as source of income, cost-effective protein rich diet and most important ingredient of the country's cultural diet. In addition, they are good source of foreign exchange earnings, next to coffee and sesame [1, 2]. Ecologically, pulses are the most important atmospheric nitrogen fixer and thus improve soil fertility and decrease the use of cost-encoring chemical nitrogenous fertilizers [3]. Thus, enhanced pulses production could create opportunities for local, national and international market and provide job opportunity and improve income of rural poor especially, women and youth [4].

Faba bean (*Vicia faba* L.) is among the most important pulse crops in the country's agricultural system. In terms of total national production, it takes the front line relative to other cultivated grain legumes [5]. For example, according to CSA [6] report, the crop covers the country's total cultivable land of 466,698 hectares with a total production of 1006751.828 tons per year which is a huge amount as compared to other legumes and pulses. It is grown for its remarkable ecological (effective nitrogen fixing ability and role in crop rotation) and nutritional values (low fat and high seed protein, carbohydrate and fiber contents) [7]. It is well grown on the cooler highlands of Ethiopia where population pressure is pronounced and other cash crops are rare [8, 9].

However, the country's production and productivity of pulses in general and faba bean in particular is below the demonstrated potential [9, 10]. For example; its average yield over the past couple of decades is nearly 2.1 t/h which is less than the average 3.7 t/h in major producing countries [11, 12]. Several yield limiting factors, including disease, unimproved cultural practices, the inherent low yielding potential of farmers' varieties, and poor soil fertility are attributed to such a significant decrease in production and productivity [13]. Shortage of improved high yielder varieties with sustainable performance and lack of awareness in using scientific methods for maintaining seed viability for longer period, one way of preserving genetic integrity, are also another pronounced constraint [14].

Continuous evaluation of the germination and performance of genetic resources, particularly improved varieties and genotypes, is an important issue and major concern in ensuring yield and other agronomic traits sustainability. Furthermore, it is critical in the design of *ex-situ* and *in-situ* conservation measures [15, 16]. In this regard, once released and recommended for use, continuous assessments of the performance and genetic diversity of the country's pulse varieties, including faba bean, are extremely poor. Lack of such information is one of the major bottlenecks in assuring their durability in performance [17]. In addition, information regarding the effects of seed storage protein on germination rate and eventually performance of Ethiopian faba bean varieties is short of what is desirable. The situation is even worse in the current scenario of unpredictable climatic change that is triggering both biotic and abiotic yield limiting factors. Thus, the present study was initiated to evaluate the effects of seed storage protein on germination rate and to assesses the performances of Ethiopian faba bean varieties collected from Holeta, Kulumsa and Sinana agricultural research systems, Ethiopia that were tested at multiple test locations. The information generated could be used as a baseline in establishing sustainable improvement and conservation of this economically important legume crop.

## 2. Materials and Methods

### 2.1. Plant Material

A total of 32 faba bean samples (31 varieties released at different times and one standard check) were collected from Holeta, Sinana and Kulumsa Agricultural Research Centers, Ethiopia where they were first released and maintained as seed source for further evaluations (Table 1). The varieties were primarily improved for yield.

### 2.2. Experiment Sites and their Description

The experiment was conducted in 2020 under rain-fed conditions at three test locations: Holeta, and Kulumsa Agricultural Research Centers, and at Elbuko center, Madda Walabu University research field, Ethiopia. Description of the locations is presented under Table 2.

### 2.3. Experimental Design

Germination test was conducted at Holeta Research Centre, Highland pulse department using distilled water, clean Petridish, and Whiteman paper. The experiment was laid out using complete random design (CRD) with three replications and ten seeds per sample in each replication.

Field experiment was laid out using a randomized complete block design (RCBD) with three replications to systematically offset field heterogeneity. Each variety was represented by 10 plants that was planted on a single row per replication using a spacing of 20 cms between plants and 50 cms between plots [18]. The varieties were assigned to the rows on random bases. NPS fertilizer was applied at a rate of 121 kg per hectare during sowing following faba bean production guideline, 2018. Weeds were controlled by hoeing and hand-weeding.

## 3. Data Collection

### 3.1. Germination Data Collection

Germination data for all study samples were collected after observing the germination of ten treated seeds per sample from the first five days to fourteen days by observing radical and plumule production according to ISTA [19]. The experiment was conducted in two replications.

### 3.2. Morphological Data Collection

Morphological data were collected using a total of 28 (22 quantitative and six qualitative) standard morphological traits following IBPGR, ICRISAT and ICARDA, [20] (Table 3). Data recording was performed on both plot and plant bases (using sampled and tagged five middle plants per row). Data recording were performed at the correct developmental stage and physiological maturity. Qualitative traits data were recorded using all the ten planted individuals for each variety over the three experimental locations (the six replications). All the quantitative traits were recorded at individual plant level in which five randomly selected and tagged plants were used per plot per replication at each site except for days to flower initiation and days to 50% flowering, which were recorded at plot level.

## 4. Data Analysis

### 4.1. Germination Data Analysis

The germination % was calculated using the following formula:

(1) $$\begin{array}{c} \text{Germination}\left(\text{\%}\right)=\frac{\text{No. of germinated seeds}}{\text{Total number of seeds sown}}\times 100\text{.} \end{array}$$

The seed germination rate was computed following Ellis and Roberts, [24]:

∑*n*/∑*D*. *n*, where *n* is the number of seeds germinated on day *D*; *D* is the number of days counted from the beginning of the test.

Mean germination time (MGT) was calculated following Ranal and Santana [25]:

(2) $$\begin{array}{c} \text{MGT}=\frac{\sum_{i=1}^{k}niti}{\sum_{i=1}^{nk}ni}, \end{array}$$

where, *ni* is the number of seeds germinated at the time *i*; *ti* is the time from the start of the experiment to the *i*^th^ observation, and *k* is the time of last germination.

### 4.2. Morphological Data Analysis

Distribution frequencies of the qualitative traits used were analyzed using Minitab® 18.1. After error variance homogeneity test using Hartley's test (F-max test) [26], analysis of variance (ANOVA) for combined data was computed following the general linear model (GLM) procedure of the SAS software (SAS version 9.0).

Estimation of environmental, genotypic and phenotypic variance components and their coefficients of variations per location and combined over locations were done following the description of Singh and Chaudhary [27]. Broad-sense heritability (H2%) per location and combined over locations, expected genetic advance (GA) under selection, assuming the selection intensity at 5%, were estimated according to Allard [28]. Similarly, genetic advance as percent of the mean was calculated as: GA (% of mean) = (GA/*m*) 9 100% where, GA = genetic advance; *m* = population mean for the trait considered.

Pairwise phenotypic and genotypic correlation coefficients were determined by using the variance and covariance components as described in Singh and Chaudhary [27] and Sharma [29]. Significances of the correlation coefficients were tested following the formula suggested by Robertson [30]; using the *t*-table at (*p* − 2) degrees of freedom, where *p* is the number of populations used in the study, at 5% and 1% level of significance.

Multivariate analyses such as principal components (PC) analysis was conducted for combined and standardized sample means using Minitab® 18.1 [31]. Population cluster analysis and pairwise generalized square distance (*D*^2^) between clusters were computed using Statistical Analysis Software (SAS 9.0).

## 5. Results

### 5.1. Germination Tests of the Varieties

The effect of seed storage on germination were measured in terms of percent germination, mean germination time and germination rate, and the result is presented under Table 4. Accordingly, the varieties showed a moderate range of germination percentage (the highest being 100% in Moyben and the smallest is 46.70.00% in Cs20-DK) and the trend showed slight concordance with the year of release. However, regardless of the large variations in years of release, the mean germination time among the tested varieties looks nearly uniform with very slight differences (10.03 in Dosha to 13 in EH940050V4). Similarly, the germination rate is nearly similar across the varieties and the local check.

### 5.2. Performance of the Varieties in Terms of the Qualitative Traits Considered

Performances of the varieties in terms of the qualitative traits considered and their distribution frequency is presented under Table 5. Accordingly, the distribution frequency of the phenotypes showed wide variations among the varieties where some of the morphotypes are rare while some others are common and proportionally distributed across the samples. For example, with regards to the distribution frequency of leaf characteristics, most varieties (63.98%) had medium sized leaves, followed by small (23.98%) and large (12.04%). Similarly, larger number of the varieties had intermediate leaf shape (63.98%) followed by those with round (20.00%), and narrow (16.02%) shapes.

Four phenotypes have been evaluated regarding pod attitude. In this regard, most of the varieties (71.99%) had erect pod attitude and the remaining showed horizontal (20.00%), mixed (7.85%) and few (0.16%) had pendent attitude. The seed size in several of the varieties (47.96%) were medium while the remaining had smaller (27.96%) and larger (24.09%) seed sizes. In addition, most of the varieties (55.97%) had flattened seed shape and the remaining had mixed (28.01%) and round (16.02%) seed shapes. With regards to seed coat color, larger number of the varieties (63.98%) had brown seed color followed by yellow (28.01%) and green (8.01%) colors.

### 5.3. Analysis of the Quantitative Trait's Mean Performance

Summary of the ranges and the means together with their standard errors, obtained on the basis of quantitative traits data combined over the three experimental locations, are shown under Table 6. In general, the faba bean varieties considered in the present study showed a wide range of variability and wide ranges between the maximum and minimum mean values in most of the quantitative traits considered. Accordingly, biomass weight per plot (*BmWPP*) and seed production efficiency (*SPE*) revealed the widest ranges with range units of 4367.34 and 3443.97, and average mean performance values of 4561.39 ± 22.12 and 69.15 ± 2.76, in that order, followed by seed yield per plot (*SYPP*) (1674.76; 1543.64 ± 16.23), and thousand seed weight (*TSW*) (315; 629.97 ± 21.12). On the other hand, three traits such as, pod width (*PoW*) (0.5), number of branches per plant (*NBPP*) (0.82), and number of seeds per pod (*NSPPo*) (0.85) showed minimum range with average mean performance values of 1.46 ± 0.02, 1.37 ± 0.04, and 2.67 ± 0.07, respectively.

### 5.4. Analysis of Variance (ANOVA)

ANOVA, computed using data combined over the three experimental locations, is presented under Table 7. Accordingly, mean square of all the traits considered showed a highly significant (*p* < 0.001) variation among the treatments (varieties). Similarly, most of the traits (fifteen of the total twenty-two) showed a significant variation over the three test locations (environment). However, only eight of the total traits revealed a significant variation for treatment (varieties)-environment interactions. A high coefficient of genetic determination (*R*^2^) was recorded for all of the traits except seed production efficiency (*SPE*) (0.59), with the highest score being 0.92 in number of seeds per pod (*NSPPo*) and days to flowering (*DTF*). Coefficient of variations (CV) is within the acceptable range for most of the traits considered with the lowest being 2.65 in days to flowering (*DTF*) and the highest being 21.38 in harvest index (*HI*).

### 5.5. Estimate of the Phenotypic and Genotypic Variance Components

Estimate of the phenotypic (*δ*^2^p) and genotypic (*δ*^2^g) variance components of the traits considered is presented under Table 8. In this regard, both the *δ*^2^p and *δ*^2^g variance estimates showed a wide range of variations in the traits considered. The minimum *δ*^2^p and *δ*^2^g (each 0.02) were recorded in pod width (*PoW*) and the maximum were (*δ*^2^p = 1072149.48 and *δ*^2^g = 964311.30) recorded in biomass weight per plot (*BmWPP*). Similarly, both phenotypic (PCV) and genotypic (GCV) coefficients of variations showed a wide range of variations in the traits considered. In this regard, PCV ranged from 2.77 in days to maturity (*DTM*) to 33.85 in seed production efficiency (*SPE*). Likewise, GCV score ranged from 2.42 in days to maturity (*DTM*) to 26.80 in seed yield per plot (*SYPP*). Half of the traits considered showed medium to high (>10%) PCV and GCV estimates. Among these, four traits such as seed production efficiency (*SPE*), biomass weight per plot (*BmWPP*), seed yield per plot (*SYPP*), and harvest index (*HI*) revealed a higher (>20%) PCV and GCV estimates. One trait, economic growth rate (*EGR*), scored higher PCV with no eventual higher GCV. The genotype-environment interaction coefficient of variation (GECV) score is detectable in most of the traits considered and showed a significant wider range of variations (0.00 in harvest index (*HI*) and biomass weight per plot (*BmWPP*) to 8.02 in seed production efficiency (*SPE*)).

In general, the difference between GCV and PCV scores in each of the traits considered is moderate (the highest being 9.88 in *SPE*) with PCV score slightly higher than the corresponding GCV score in all the traits considered.

### 5.6. Estimates of Heritability in Broad Sense and Genetic Advance

Estimates of heritability and genetic advance for the traits considered is presented under Table 8. In this regard, estimate of heritability in broad sense (H^2^%) revealed a wide range of variations (29.33 in leaf length (*LL*) to 92.61% in economic growth rate (*EGR*)) among all the traits considered. According to Singh [32]; high heritability of a character (≥80%) could warrant targeted selection since it implicates close correspondence between the genotype and the phenotype due to the relatively small contribution of the environment factors. Whereas, for characters with low heritability (40% or less), selection may be considerably difficult or impractical due to the masking effect of the environment. Considering this bench-mark, most of the traits (13 or 59.09%) scored high heritability estimates and thus important for further selection work.

Genetic advance under selection (GA) refers to the improvement of characters in genotypic value for the new population compared with the base population under one cycle of selection at a given selection intensity [32]. In this regard, estimates of genetic advance (GA) among the traits revealed a wider variation (0.27 in pod width (*PoW*) to 1914.75 in biomass weight per plot (*BmWPP*). Similarly, considerably higher genetic advance as percent of mean (GAM) was recorded in seed yield per plot (*SYPP*) (52.36%), biomass weight per plot (*BmWPP*) (41.98%), harvest index (*HI*) (39.68%) and economic growth rate (*EGR*) (38.98%).

### 5.7. Analysis of Correlation Coefficients

The extents of pair-wise genotypic (above diagonal) and phenotypic (below diagonal) correlation coefficients between the traits considered is presented under Table 9. In this regard, most of the traits considered showed a significant genotypic and phenotypic pair-wise associations though some showed a non-significant association.

Yield and yield related traits, an important aspect in food crops, had a significant association with most of the traits considered. For example, seed yield per plot (*SYPP*), a direct reflection of seed yield per hectare, showed a highly significant (*p* < 0.001) genotypic and phenotypic association with the remaining 13 and 16 traits, respectively.

### 5.8. Principal Components and Cluster Analyses

Both principal components analysis (PCA) and clustering were conducted using pooled standardized data of the 22 quantitative traits. Accordingly, the first six principal axes (eigen value ≥1.00) in PCA accounted for 86.00% of the total variation (Table 10). The first principal component (PC1) accounted for 32.00% of the total variation. The variations in this component were largely contributed by days to flowering (*DTF*), leaf length (*LL*) and internode length (*IL*) with factor loadings of 0.32 (the first two) and 0.31. The second PC axis accounted for 24.00% of the total variation and differentiated the varieties largely on the bases of seed filling period (*SFP*) (0.30), number of seeds per pod (*NSPPo*) and leaf area (*LA*) (each 0.29), number of seeds per plant (*NSPP*) (0.28), and seed yield per plot (*SYPP*) (0.27). The third and fourth PCs axis contributed 11.00% and 7.00% of the total variations, respectively with high contributing factor loadings from harvest index (*HI*; 0.58), number of seeds per plant (*NSPP*; 0.37), seed producing efficiency (*SPE*) and leaf width (*LW*) (each −0.33), height to the first podding node (*PHFPo*), plant height (*PH*) (each −0.31). The fifth and sixth PCs axis each contributed almost the same variations (5.00% and 6.00% of the total variations) and had high factor loadings from pod width (*PoW*) (0.54), pod length (*PoL*) (0.43), leaf width (*LW*) (−0.36), and leaf area (*LA*) (−0.35) (Table 10).

**Figure 1:**
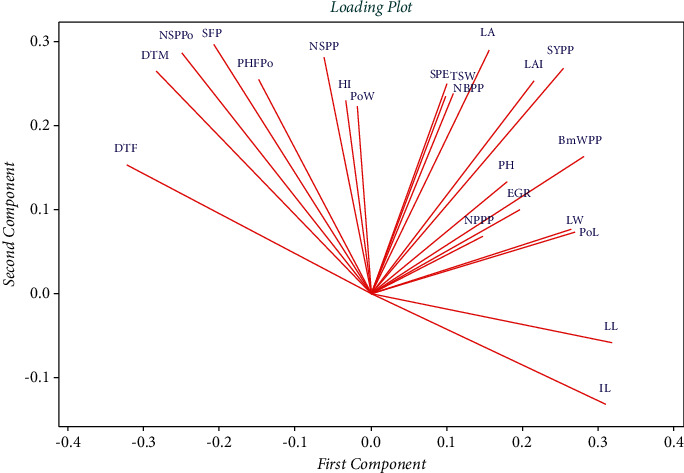
PCA loading plot showing the extents and direction of association among the 22 quantitative traits used in the present study.

Similarly, PCA loading plot showed a very strong and close correlation among such traits as seed yield per plot (*SYPP*), number of seeds per plant (*NSPP*), biomass weight per plant (*BwPP*), plant height (*PH*) and thousand seed weight (*TSW*). Moreover, there exist a strong positive correlation among the remaining traits except the association between days to flowering (*DTF*) *versus* inter-node length (*IL*), and leaf length (*LL*) (Figure 1).

**Figure 2:**
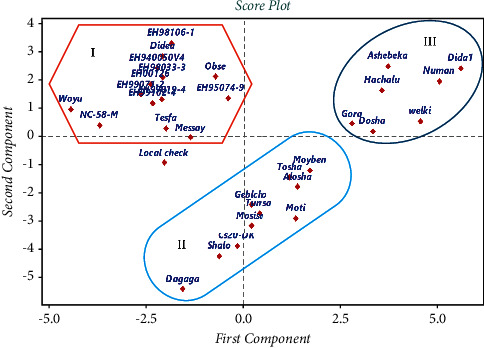
PCA Score plot showing the clustering pattern of the faba bean varieties considered in the present study.

**Figure 3:**
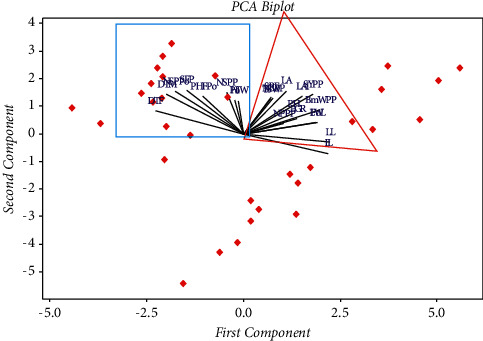
PCA Biplot showing the association between the 22 traits used and the 31 varieties considered; the red dots represent faba bean varieties used in the present study.

PCA score plot revealed that the entire varieties were grouped roughly into three groups excluding the local check that appeared alone. The grouping pattern weakly followed the year of release or pedigree of their ancestral line. Thus, the result showed that several varieties released at different times were clustered together and varieties released during the same years were placed under different groups (Figure 2). Likewise, PCA biplot revealed that the varieties have made strong layover on the second component contribution from several of the traits considered (Figure 3).

**Figure 4:**
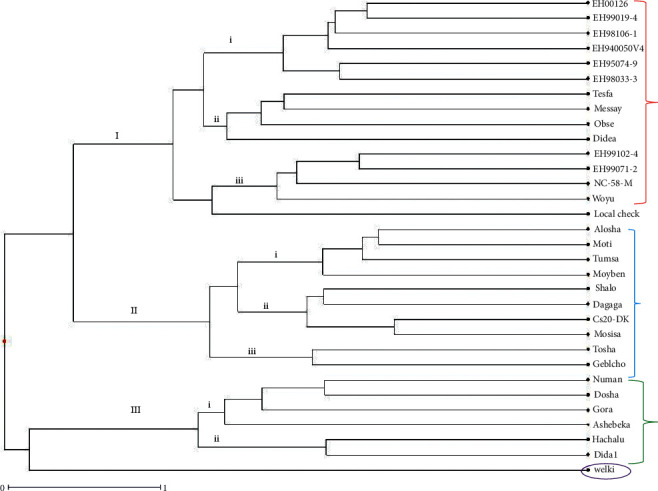
Clustering of the 32 faba bean samples evaluated in the present study.

Clustering of the entire 32 samples (31 varieties and one local check) revealed roughly three major clusters (Figure 4). The first cluster (*I*) contained the largest number (15 of the total 32 samples) of varieties which were sub-divided into three sub-clusters (i, ii, and iii). The second cluster (II) contained ten varieties which were again sub-clustered into three groups (i, ii, and iii). The third cluster contained only six varieties which appeared in two sub-clusters. One variety, Welki appeared as monophyletic (Figure 4; Supplementary Table 1).

Clusters II *vs* III had the largest inter-cluster distance (91.27) followed by I *vs* III (49.34) and I *vs* II (20.14). on the other hand, varieties in cluster II and III had the largest intra-cluster distance (each 4.28) as compared to varieties in cluster I, which had intra-cluster distance of 2.89 (Table 11).

## 6. Discussions

Faba bean is largely cultivated in the highland areas of the country where population density, land degradation and shortage of farm-land are major concerns. It is one of the cheap sources of protein and crops of food security in Ethiopian diet. In the present study, performance of a total of 32 faba bean samples (31 varieties and one local check) have been tested at multiple locations and implications of the results obtained are presented below:

### 6.1. Effects of Seed Storage Time on Faba Bean Germination

Life processes of a given seed is partly dependent on the storage conditions and thus, there is no defined demarcation of life-time for a given seed [33]. Seed death is commonly a gradual process and sometimes confusing to detect since some seeds radically decrease their germination rate and become less vigorous or worthless long before the actual death, especially under field condition [34]. In this regard, the present study revealed a good and promising germination percentage, germination rate and mean germination time in most of the varieties considered. However, there are detectable variations among the varieties with regards to their germination percentage that seems concordant with storage time. Accordingly, most of the recently released varieties showed higher germination percentage as compared to those older varieties which could be attributed to storage conditions such as storage temperature, external environmental conditions, and genetic factors such as seed moisture content. There are similar reports suggesting variation in germination rate among different genotypes depending on their storage duration and eventually storage conditions [35, 36].

### 6.2. Performances of the Qualitative and Quantitative Traits

The present study revealed a varied performance, expressed as frequency distribution, in most of the qualitative traits considered. Similar result has been reported by Thomas et al., [37]. Such wide performance variation could be attributed to maintained genetic variations among the tested varieties that is eventually useful for further production and selection breeding activities. Similarly, the mean performance values of the 22 quantitative morphological traits considered revealed a wide range of variation suggesting wide variability in both phenotypic and genotypic values that are useful to identify promising varieties for yield potential and quality. Moreover, the tested varieties revealed statistically significant differences in most of the traits revealing the presence of substantial variation which offers a room for further adaptation and a good opportunity for further improvement through selection breeding. Similar result has been reported by Mulugeta et al., [14]; Alghamdi [38]; Sharifi [39]; Gadissa et al., [16] and Ammar et al. [40]. The effect of environment, variety-environment interaction, blocking and replication showed no significant variation among the varieties in most of the traits considered suggesting the consistent genetic performance of the variations and smaller environmental effects.

Yield and yield related trait's performance are very essential and several breeding attempts are directed towards the improvement and maintenance of those traits. In this regard, all the tested varieties revealed a statistically significant (*p* < 0.001) variation where Dida1 showed a good yield performance with the highest average seed yield of 2459.73 kg/ha, followed by Welki (2394.48 kg/ha), Hachalu (2243.92 kg/ha), Ashebeka (2224.72 kg/ha) and Obse (2114.94 kg/ha). The result is in line with the reports of Yirga and Zinabu [41]; Mulugeta et al., [14]; and Kubure et al. [42]. The result indicates relative stable performance of the varieties regardless of their year of release. On the contrary, Degaga (784.97 kg/ha) and Shalo (965.51 kg/ha) showed a reduced performance that might be attributed to their reduced adaptive potential to environmental changes and to different ecological conditions and thus their less rewarding nature to further use in breeding and conservation. There are reports supporting the lower and non-stable performance of these varieties under different environmental conditions and over years [43].

### 6.3. Implications of the Patterns of Phenotypic and Genotypic Variations

Patterns of variation in the genotypic and phenotypic performances are the major tools to measure the variability that exists in a given population [44]. In this regard, the wide range of variations scored in both the phenotypic and genotypic coefficients of variations (PCV and GCV) indicate stable performance of the varieties and gradual accumulation of genetic variability that is useful for further targeted selection and breeding of faba bean varieties. The slightly higher PCV estimate over the corresponding GCV values in most of the traits and the relative narrow gap between them indicates the small environmental effects on the traits that once again assure the genetic base of the variations which is expected in genotypes that are under breeding scheme for so long. There have been similar reports by Yirga and Zinabu [41]; Mulugeta et al., [14]; and Kubure et al. [42] on different faba bean varieties. According to Sharifi [45]; sufficiently high heritability value shows minimal influence of environment response on detectable traits. In this regard, the present study revealed that larger number of the traits considered (59.09%) had high broad sense heritability value (>80%) suggesting their relative importance in further selection breeding of faba bean genotypes. Similar results have been reported by Alghamdi [38] and Million and Habtamu [46] for several of the traits and varieties.

Estimate of genetic advance (GA) is important to improve genotypic value of a given character in the base population under one cycle of selection at a given selection intensity. However, it is more feasible if coupled with heritability and other variance components [32]. Thus, higher estimates of heritability along with high genetic advance (GA) and genetic coefficient of variation (GCV) provide good scope for further stability and improvement through phenotypic selection [27, 44]. In this view, four traits such as seed producing efficiency (*SPE*), biomass weight per plot (*BmWPP*), seed yield per plot (*SYPP*), and harvest index (*HI*) are very important.

### 6.4. Patterns of Association between the Traits

Pairwise correlation coefficient analysis determines the magnitude and degree of relationship between two traits. The association could be due to genotypic (linkage between genes) or pleiotropic gene effect, or due to environmental correlation, or both [47, 48]. With this view, the highly significant association between seed yield and several other traits offer an indirect opportunity for maintaining yield through improving those traits so that breeders could use them as selection criterion. The result is in close agreement with Alghamdi [38]; Gemechu et al., [49, 50] and Mulugeta et al., [14].

### 6.5. Patterns of Grouping in the Varieties

Principal component analysis (PCA) is important in understanding the sources of variation among the study samples and to find out the characters which accounted more to the total variation. In the present study, the first six principal components (Eigen value ≥1.00) accounted nearly 86.00% of the total variations and score plot distinguished the varieties into three clusters implying significant amount of genetic variability among the tested varieties and thus good opportunity in maintaining the varieties and using them for targeted breeding programs. Several of the traits considered contributed for the largest variation suggesting that selection based on these morphological traits may be effective. Likewise, biplot and loading plot graphs demonstrated a positive association among the traits and varieties showing their relative importance in improving the varieties.

Cluster analysis has a power to tell us how samples are genetically similar to each other or different from each other. In this regard, the tested varieties were grouped into three major clusters. However, the clusters had a considerable inter-cluster distance (the maximum and minimum being *D*^2^ = 91.27, and 20.14, respectively) indicating that faba bean varieties in each cluster are still maintained sufficient variability and thus further selection between the clusters could bring high genetic gain for the character of interest.

## 7. Conclusions

Current faba bean production in Ethiopia is very much less than the actual potential of the country. One of the reasons is lack of quality seeds with sustainable performance under varying conditions. In this regard, the present study generated a baseline information that could be used to conduct sustainable faba bean breeding and improvement. The wide range of variation in all the qualitative traits considered signals the importance of those traits for selection breeding. Similarly, the significant variations in most of the quantitative traits considered could be exploitable in improvement of the crop at large. The high genetic advance coupled with heritability and genotypic coefficient of variation observed in some of the traits could reveal their high importance for selection breeding. This is because larger extents of heritability coupled with genetic advance and genotypic coefficient of variations is most useful to indicate the amount of genetic improvement that would result from selection of individual genotypes. Grouping of the genotypes showed high difference among the clusters suggesting large difference in seed performance, morphological traits and yield or in genetic background between aged seeded varieties and the recent ones. However, further research involving advanced molecular markers needs to be carried out to clearly indicate the extents of genetic diversity in the varieties and thus, their performance.

## Figures and Tables

**Table 1:** Detailed description of the faba bean varieties used in the present study.

S/N	Variety^*∗*^	Pedigree	Center of release and maintenance^*∗∗*^	Year of release
1	Moti	EH95078-6	HARC	2006
2	Geblcho	EH96009-1	HARC	2006
3	Dosha	COLL 155/00-3	HARC	2009
4	Dida1	ICB2717-1xR-878-3	KARC	2014
5	Gora	EH91026-8-2xBPL44-1	KARC	2013
6	Hachalu	EH00102-4-1	HARC	2010
7	Dagaga	R-878-3	HARC	2002
8	Tumsa	EH99051-3	HARC	2010
9	Ashebeka	EH01075-4	KARC	2015
10	Welki	EH96049-2	KARC	2008
11	Alosha	EH20066-5	SARI	2017
12	Tosha	EKLS/CSR02022-1-1	SARI	2019
13	Moyben	EH04629-1	SARI	2019
14	Shalo	EH011-22-1	SARI	1999
15	Mosisa	EH99077	SARI	2013
16	Numan	EH56065-3	KARC	2016
17	Cs20-DK	CS-20-DK	HARC	1978
18	Messay	4A12050x74TA236	HARC	1996
19	Obse	EH95073-1	HARC	2007
20	Tesfa	75TA2626-1-2-1	HARC	2007
21	Woyu	WAYU 89-5	HARC	2002
22	Didea	EH06067-3	HARC	2016
23	NC-58-M	NC-58-M	HARC	2010
24	EH99071-2	EH99071-2	HARC	2006
25	EH99019-4	EH99019-4	HARC	2005
26	EH940050V4	EH940050V4	HARC	2004
27	EH98033-3	EH98033-3	HARC	2006
28	EH99102-4	EH99102-4	HARC	2007
29	EH00126	EH00126	HARC	2006
30	EH95074-9	EH95074-9	HARC	2006
31	EH98106-1	EH98106-1	HARC	2007
32	Standard check		HARC	

^*∗*^The varieties have already been registered in the national pulse catalogue; ^*∗∗*^HARC = holeta agricultural research center; KARC = kulumsa agricultural research center; SARI = sinana agricultural research institute; Both HARC and KARC are under Ethiopian institute of agricultural research (EIAR); SARI is under Oromia agricultural research institute (OARI).

**Table 2:** Description of the three experiment locations used in the present study.

Parameters		Test locations		
		Holeta	Kulumsa	Elbuko
Distance from the capital Addis Ababa in km and direction		40; SW	170; S	563; SE
Altitude (m.a.s.l)		2400	2200	2406
Temperature (min and max) in °C		6 and 22	10 and 22	10 and 25
Average annual rainfall in mm		1144	800	918
Soil texture		Clay	Clay	Clay
Global positioning	Latitude	9^o^00′06.78″N	8^o^20′33.05″N	0709′02.99″N
	Longitude	38^o^3′45.35″E	39^o^10′25.03″E	4042′48.13″E

SW = Southwest; *S* = South; SE = Southeast.

**Table 3:** Standard qualitative and quantitative agro-morphological traits used for data recording along with their description.

S/N	Traits	Description	Remark
	Qualitative		
1	Leaf size	Scored by visual observation on each plot by considering three leaf sizes: small (1), medium (2), and large (3)	
2	Leaf shape	Scored based on visual observation from the plot by considering shapes such as narrow (1), intermediate (2), and rounded (3)	
3	Pod attitude	Recorded as erect (1), horizontal (2), pendent (3) and mixed (4	
4	Seed shape	Recorded based on visual observation of the seeds and determined as oblong (1), semi spherical (2), semi oval (3)	
5	Seed size	Scored as small (1), medium (2) and large (3)	
6	Seed coat color	Recorded based on visual observation of the seed coat as green (1), yellow (2) and (brown)	

	*Quantitative*		
1	Leaf length (LL) (cm)	Average length of three leaves taken from bottom, middle and top of a plant and measured from the base to the tip of the leaf	
2	Leaf width (LW) (cm)	Average width measured at the widest point of the central leaflet of the trifoliate leaf taken from top, middle and bottom of the plant	
3	Leaf area (LA) (cm^2^)	Average area of three leaves calculated using Peksen [21] model (LA = 0.919 + 0.682 LW)	
4	Leaf area index (LAI)	Area covered by plant and calculated as; LAI = leaf area × number of leaves per plant	
5	Number of branches per plant (NBPP)	Count of branches from basal and mediated nodes per plant	
6	Number of pods per plant (NPPP)	The average numbers of pods counted from samples of five plants per row	
7	Number of seeds per pod (NSPPo)	The total number of seeds per plant divided by the total number of pods on the same plant and averaged over five plants per row	
8	Number of seeds per plant (NSPP)	The total count of seeds per plant	
9	Plant height (PH) (cm)	The average height of five plants per row measured at physiological maturity	
10	Height to the first podding node (PHFPo) (cm)	The average height of five plants measured from ground to the first pod bearing nodes at physiological maturity	
11	Pod length (PoL) (cm)	Average exterior distance of fully matured pod from the pod apex to the peduncle measured from top, middle and bottom plant parts	
12	Pod width (PoW)	Average width of three pods per plant for the five selected plants measured at the center of pod using a caliper	
13	Internode length (IL)	Average length of three internodes per plant in the five selected plants	
14	Days to flowering (DTF)	Number of days from planting to 50% flowering	
15	Days to maturity (DTM)	Number of days from sowing to the stage when 90% of the plants in a row reached physiological maturity	
16	Seed filling period (SFP)	The number of days from flowering to maturity (number of days to maturity minus number of days to flowering)	
17	Thousand seed weight (TSW) (g)	The weight of thousand seeds taken randomly from the harvest seed lots of each plot	
18	Seed yield per plot (grain yield) (SYPP) (g)	Grain yield in gram from the harvestable plot area. It was adjusted to 10% moisture level to give adjusted yield. This value was converted into kg/ha and used for analysis.	
19	Seed production efficiency (SPE)	Seed filling duration divided by duration of vegetative period and then multiplied by grain yield	
20	Biomass weight per plot (BmWPP) (g/m^2^)	Weight of whole above ground plant parts on the row harvested, sun dried and weighted	
21	Harvest index (HI)	Ratio of grain yield to above ground biological yield calculated following Manfred [22] and Rkmhay [23]	
22	Economic growth rate (EGR)	The ratio of seed weight per row to seed filling duration times 100	

**Table 4:** Percent of germination, mean germination time and germination rate of the tested faba bean genotypes.

Variety	Year of release	Germination %	Mean germination time	Germination rate/day
Moti	2006	83.30	10.37	0.10
Geblcho	2006	80.00	10.60	0.10
Dosha	2009	96.70	10.03	0.10
Dida1	2014	93.30	10.63	0.09
Gora	2013	90.00	11.33	0.09
Hachalu	2010	73.30	12.33	0.10
Dagaga	2002	76.60	10.73	0.09
Tumsa	2010	84.00	10.44	0.08
Ashebeka	2015	81.00	11.25	0.10
Welki	2008	78.00	10.66	0.09
Alosha	2017	90.00	11.31	0.10
Tosha	2019	88.00	10.36	0.08
Moyben	2019	100.00	10.20	0.09
Shalo	2000	70.00	10.39	0.09
Mosisa	2013	80.00	10.73	0.09
Numan	2016	90.00	10.43	0.10
Cs20-DK	1978	46.70	10.14	0.08
Messay	2004	67.00	11.77	0.09
Obse	2015	56.70	12.50	0.09
Tesfa	2007	53.33	11.45	0.08
Woyu	2003	66.70	12.43	0.09
Didea	2016	88.00	11.22	0.10
NC-58-M	2010	66.60	11.63	0.08
EH99071-2	2006	63.30	12.00	0.09
EH99019-4	2005	53.70	10.82	0.09
EH940050V4	2004	51.70	13.00	0.08
EH98033-3	2006	63.30	11.10	0.08
EH99102-4	2007	76.70	11.00	0.09
EH00126	2006	60.00	10.87	0.09
EH95074-9	2006	63.33	12.67	0.08
EH98106-1	2007	63.30	10.50	0.08
Local check	2018	90.00	11.34	0.09

**Table 5:** Qualitative traits used in the study along with their phenotype, phenotype code and number or frequency of genotypes.

Traits	Phenotype	Phenotype code	No of genotypes^*∗*^
Leaf size	Small	1	446 (23.98)
	Medium	2	1190 (63.98)
	Large	3	224 (12.04)
Leaf shape	Narrow	1	298 (16.02)
	Intermediate	2	1190 (63.98)
	Rounded	3	372 (20.00)
Pod attitude	Erect	1	1339 (71.99)
	Horizontal	2	372 (20.00)
	Pendent	3	3 (0.16)
	Mixed	4	146 (7.85)
Seed size	Small	1	520 (27.96)
	Medium	2	892 (47.96)
	Large	3	448 (24.09)
Seed shape	Flattened	1	1041 (55.97)
	Round	2	298 (16.02)
	Mixed	3	521 (28.01)
Seed coat color	Green	1	149 (8.01)
	Yellow	2	521 (28.01)
	Brown	3	1190 (63.98)

^*∗*^The total count of genotypes showing the phenotype; numbers in parenthesis shows % of genotypes possessing a phenotype.

**Table 6:** Performance of the quantitative traits expressed in mean's, range and range-unit.

Traits^*∗*^	Trait mean ± SE	Range (min to max)	Range unit
*LL*	7.51 ± 1.20	6.33–8.96	2.63
*LW*	3.99 ± 0.52	2.95–4.99	2.04
*LA*	25.42 ± 3.12	12.47–32.95	20.48
*LAI*	186.6 ± 28.54	136.04–245.68	109.64
*PoL*	6.89 ± 1.21	5.68–8.20	2.52
*PoW*	1.46 ± 0.02	1.26–1.76	0.50
*IL*	4.48 ± 1.87	3.79–5.11	1.14
*PHFPo*	33.5 ± 3.22	28.06–40.00	11.94
*PH*	106.87 ± 11.12	90.81–123.55	32.74
*NBPP*	1.37 ± 0.04	0.99–1.81	0.82
*NPPP*	23.47 ± 1.24	18.57–30.56	11.99
*NSPP*	61.97 ± 4.67	45.71–78.97	33.26
*NSPPo*	2.67 ± 0.07	2.21–3.06	0.85
*DTF*	58.34 ± 3.34	53.17–64.83	11.66
*DTM*	128.44 ± 6.19	120.00–138.44	18.44
*SFP*	69.15 ± 2.76	63.50–78.20	14.70
*SPE*	2422.07 ± 65.33	1031.00–4474.97	3443.97
*TSW*	629.97 ± 21.12	484.67–799.67	315.00
*EGR*	52.08 ± 3.34	34.60–77.14	42.54
*BmWPP*	4561.39 ± 22.12	2890.63–7257.97	4367.34
*SYPP*	1543.64 ± 16.23	784.97–2459.73	1674.76
*HI*	34.37 ± 5.29	23.54–56.59	33.05

^*∗*^LL = leaf length; LW = leaf width; LA = leaf area; LAI = leaf area index; NBPP = number of branches per plant; NPPP = number of pods per plant; NSPPo = number of seeds per pod; NSPP = number of seeds per plant; PH = plant height; PHFPo = height to the first podding node; PoL = pod length; PoW = pod width; IL = internode length; DTF = days to flowering; DTM = days to maturity; SFP = seed filling period; TSW = thousand seed weight; SYPP = seed yield per plot; SPE = seed production efficiency; BmWPP = biomass weight per plot; HI = harvest index; EGR = economic growth rate.

**Table 7:** Analysis of Variance (ANOVA) for the 22 quantitative traits evaluated using the 32 faba bean samples at the three test locations.

Variables	Envt. (2)	Rep (Envt.) (2)	Block(Rep) (1)	Trt (30)	Trt^*∗*^Envt. (62)	MSE (92)	CV (%)	*R* ^2^
*LL*	1.77^*∗*^	1.29	0.03	1.61^*∗∗∗*^	1.16^*∗∗∗*^	0.57	10.01	0.75
*LW*	0.97^*∗∗∗*^	0.13	0.02	1.25^*∗∗∗*^	0.18^*∗∗∗*^	0.11	8.15	0.87
*LA*	30.52^*∗∗*^	0.05	7.36	94.47^*∗∗∗*^	11.14^*∗∗∗*^	4.78	8.59	0.89
*LAI*	993.75	647.02	1545.64	4382.33^*∗∗∗*^	620.51	500.21	11.98	0.79
*PoL*	0.06	0.99	2.14^*∗*^	1.91^*∗∗∗*^	0.44	0.33	8.31	0.81
*PoW*	0.07^*∗*^	0.04	0.01	0.11^*∗∗∗*^	0.02	0.02	9.47	0.75
*IL*	1.94^*∗∗∗*^	0.06	0.53	0.34^*∗∗∗*^	0.13	0.1	7.09	0.79
*PHFPo*	20.22^*∗*^	0.43	2.39	37.10^*∗∗∗*^	6.34	5.21	6.82	0.83
*PH*	125.83^*∗∗*^	3.72	30.31	285.23^*∗∗∗*^	28.43	24.53	4.63	0.83
*NBPP*	0.01	0.03	0.15	0.31^*∗∗∗*^	0.06	0.06	17.45	0.73
*NPPP*	64.96^*∗∗∗*^	14.85	24.12^*∗∗*^	43.10^*∗∗∗*^	6.98	5.49	9.99	0.8
*NSPP*	364.43^*∗∗*^	164.41^*∗*^	4.37	325.75^*∗∗∗*^	80.35	49.16	11.31	0.79
*NSPPo*	0.09^*∗∗*^	0.03	0.07	0.19^*∗∗∗*^	0.05^*∗∗∗*^	0.02	5.12	0.92
*DTF*	18.87^*∗∗∗*^	0.23	8.33	24.27^*∗∗∗*^	6.13^*∗∗∗*^	2.39	2.65	0.92
*DTM*	270.47^*∗∗∗*^	9.35	8.01	75.86^*∗∗∗*^	18.01	12.59	2.76	0.91
*SFP*	77.36^*∗∗∗*^	9.63	56.71^*∗*^	46.09^*∗∗∗*^	12.49^*∗∗∗*^	5.64	3.43	0.88
*SPE*	1565460.3	2147049.5	1424726.3	4146047.60^*∗∗*^	2040626.3	19516	57.67	0.59
*TSW*	1838.02	1378.57	764.6	40953.14^*∗∗∗*^	3429.99^*∗*^	2018.39	7.13	0.89
*EGR*	167.02^*∗∗*^	61.23	10.65	615.02^*∗∗∗*^	50.17^*∗*^	33.09	11.05	0.89
*BmWPP*	146825.4	128374.8	2220361.2	6130279.1^*∗∗∗*^	431070.8	815713.5	19.8	0.78
*SYPP*	242702.23	48456.4	168864.47	1096137.17^*∗∗∗*^	110250.35	95135.76	19.98	0.83
*HI*	178.4^*∗*^	8.89	455.38^*∗∗*^	351.90^*∗∗∗*^	43.67	53.99	21.38	0.76

Description of the traits is presented under Table 2; envt. = environment; rep (envt.) = replication within environment; block (rep) = block within replication; Trt = treatment (varieties); Trt^*∗*^envt. = treatment-environment interaction; MSE = mean square error; CV = coefficient of variation; *R*^2^ = coefficient of genetic determination.

**Table 8:** Estimate of the variance components in the 22 quantitative traits used to evaluate the performance of faba bean varieties.

Traits	*δ* ^2^e	*δ* ^2^g	*δ* ^2^ge	*δ* ^2^p	GCV (%)	PCV (%)	GECV (%)	Hb (%)	GA	GAM
*LL*	0.00	0.08	0.30	0.27	3.77	6.97	7.24	29.23	0.31	4.19
*LW*	0.01	0.24	0.04	0.27	12.28	13.02	5.01	88.89	0.95	23.80
*LA*	0.31	13.39	3.23	15.25	14.40	15.36	7.07	87.82	7.05	27.74
*LAI*	3.54	618.70	60.04	722.12	13.33	14.40	4.15	85.68	47.34	25.37
*PoL*	0.00	0.25	0.05	0.32	7.26	8.23	3.25	77.72	0.91	13.15
*PoW*	0.00	0.02	0.00	0.02	9.69	10.54	2.17	84.51	0.27	18.31
*IL*	0.03	0.04	0.02	0.06	4.46	5.54	3.16	64.86	0.33	7.39
*PHFPo*	0.22	5.20	0.62	6.26	6.81	7.47	2.35	83.09	4.27	12.76
*PH*	1.52	41.57	2.16	46.31	6.03	6.37	1.38	89.77	12.56	11.75
*NBPP*	0.00	0.04	0.00	0.05	14.60	16.43	3.26	78.95	0.37	26.67
*NPPP*	0.81	6.04	0.73	7.20	10.47	11.43	3.64	83.87	4.63	19.72
*NSPP*	3.22	49.61	15.83	63.00	11.37	12.81	6.42	78.75	12.85	20.74
*NSPPo*	0.00	0.03	0.01	0.04	6.49	7.17	3.75	81.82	0.32	12.06
*DTF*	0.19	3.04	1.89	4.06	2.99	3.45	2.36	74.85	3.10	5.32
*DTM*	3.94	9.67	2.74	12.67	2.42	2.77	1.29	76.33	5.59	4.35
*SFP*	0.95	5.65	3.43	7.73	3.44	4.02	2.68	73.06	4.18	6.04
*SPE*	0.00	334374.50	37764.40	672004.05	23.87	33.85	8.02	49.76	838.63	34.62
*TSW*	0.00	6057.00	704.30	6620.37	12.35	12.92	4.21	91.49	153.05	24.30
*EGR*	1.67	104.79	8.66	113.15	19.66	20.42	5.65	92.61	20.25	38.89
*BmWPP*	0.00	964311.30	0.00	1072149.48	21.53	22.70	0.00	89.94	1914.75	41.98
*SYPP*	2069.60	171120.50	8098.50	189495.57	26.80	28.20	5.83	90.30	808.21	52.36
*HI*	2.02	51.08	0.00	59.30	20.79	22.40	0.00	86.14	13.64	39.68

Description of the traits is presented under Table 2; *δ*^2^e = environmental variance; *δ*^2^g = genotypic variance; *δ*^2^ge = variance due to genotype environment interaction; *δ*^2^p = phenotypic variance; GCV = genotypic coefficients of variation; PCV = phenotypic coefficients of variation; GECV = genotype-environment interaction coefficients of variation; Hb = heritability in broad sense; GA = genetic advance; GAM = genetic advance as a percent of traits means.

**Table 9:** Combined pair-wise genotypic (above diagonal) and phenotypic (below diagonal) correlation coefficient of the 22 quantitative traits considered in the study.

Variable	*LL*	*LW*	*LA*	*LAI*	*PoL*	*PoW*	*IL*	*PHFPo*	*PH*	*NBPP*	*NPPP*
*LL*	1	0.54^*∗∗*^	0.32	0.39^*∗*^	0.61^*∗∗∗*^	−0.18	0.65^*∗∗∗*^	−0.41^*∗*^	0.46^*∗*^	0.20	0.19
*LW*	0.33^*∗∗∗*^	1	0.68^*∗∗∗*^	0.44^*∗*^	0.49^*∗∗*^	0.04	0.45^*∗∗*^	−0.04	0.27	0.34	0.26
*LA*	0.26^*∗∗*^	0.59^*∗∗∗*^	1	0.71^*∗∗∗*^	0.46^*∗∗*^	0.23	0.04	0.37^*∗*^	0.44^*∗*^	0.52^*∗∗*^	0.06
*LAI*	0.32^*∗∗∗*^	0.35^*∗∗∗*^	0.56^*∗∗∗*^	1	0.50^*∗∗*^	0.28	0.29	0.21	0.65^*∗∗∗*^	0.39^*∗*^	0.10
*PoL*	0.35^*∗∗∗*^	0.32^*∗∗∗*^	0.31^*∗∗∗*^	0.35^*∗∗∗*^	1	0.25	0.44^*∗*^	−0.24	0.28	0.17	0.11
*PoW*	−0.06	0.07	0.18	0.18^*∗*^	0.05	1	−0.25	0.09	−0.14	0.04	0.06
*IL*	0.23^*∗∗*^	0.29^*∗∗∗*^	0.02	0.14	0.32^*∗∗∗*^	−0.17^*∗*^	1	−0.49^*∗∗∗*^	0.26	−0.00	0.38^*∗*^
*PHFPo*	−0.23^*∗*^	0.01	0.27^*∗∗∗*^	0.16^*∗*^	−0.15^*∗*^	0.09	−0.38^*∗∗∗*^	1	0.26	0.34	−0.27
*PH*	0.28^*∗∗∗*^	0.25^*∗∗∗*^	0.35^*∗∗∗*^	0.50^*∗∗∗*^	0.21^*∗∗*^	−0.03	0.17^*∗*^	0.24^*∗∗*^	1	0.24	−0.09
*NBPP*	0.19^*∗∗*^	0.18^*∗*^	0.33^*∗∗∗*^	0.24^*∗∗*^	0.11	−0.00	−0.04	0.14	0.16^*∗*^	1	0.31
*NPPP*	0.16^*∗*^	0.20^*∗∗*^	0.04	0.03	0.10	−0.01	0.18^*∗*^	−0.25^*∗*^	−0.11	0.24^*∗∗*^	1
*NSPP*	−0.08	0.04	0.15^*∗*^	0.05	0.00	0.24^*∗∗*^	−0.17^*∗*^	0.07	−0.09	0.26^*∗∗∗*^	0.68^*∗∗∗*^
*NSPPo*	−0.35^*∗∗∗*^	−0.21	0.18^*∗*^	0.05	−0.15^*∗*^	0.39^*∗∗∗*^	−0.49^*∗∗∗*^	0.47^*∗∗∗*^	−0.04	0.08	−0.21^*∗*^
*DTF*	−0.51^*∗∗∗*^	−0.34^*∗∗*^	−0.06	−0.26^*∗∗∗*^	−0.35^*∗∗∗*^	0.10	−0.48^*∗∗*^	0.47^*∗∗∗*^	−0.26^*∗∗∗*^	−0.04	−0.24^*∗∗∗*^
*DTM*	−0.54^*∗∗∗*^	−0.28^*∗∗∗*^	0.05	−0.09	−0.35^*∗∗∗*^	0.24^*∗∗∗*^	−0.44	0.52^*∗∗∗*^	−0.09	0.10	−0.15^*∗*^
*SFP*	−0.3^*∗∗*^	−0.25^*∗∗∗*^	0.13	0.07	−0.24^*∗*^	0.30^*∗∗∗*^	−0.36^*∗∗∗*^	0.43^*∗∗∗*^	−0.02	0.16^*∗*^	−0.11
*SPE*	0.05	0.29^*∗∗∗*^	0.29^*∗∗∗*^	0.27^*∗∗*^	0.11	0.13	−0.02	0.19^*∗∗*^	0.19^*∗∗*^	0.14^*∗*^	0.03
*TSW*	0.12	0.10	0.31^*∗∗∗*^	0.21^*∗∗*^	0.35^*∗∗∗*^	0.29^*∗∗∗*^	0.05	−0.04	0.08	0.31^*∗∗∗*^	0.12
*EGR*	0.22^*∗∗*^	0.19^*∗*^	0.06	0.16^*∗*^	0.31^*∗∗∗*^	0.09	0.29^*∗∗∗*^	−0.30^*∗∗∗*^	0.04	0.25^*∗∗∗*^	0.57^*∗∗∗*^
*BmWPP*	0.32^*∗∗∗*^	0.47^*∗∗∗*^	0.38^*∗∗∗*^	0.56^*∗∗∗*^	0.31^*∗∗∗*^	0.23^*∗∗*^	0.29^*∗∗∗*^	0.07	0.36^*∗∗∗*^	0.24^*∗∗∗*^	0.16^*∗*^
*SYPP*	0.34^*∗∗∗*^	0.34^*∗∗∗*^	0.48^*∗∗∗*^	0.56^*∗∗∗*^	0.37^*∗∗∗*^	0.15^*∗*^	0.26^*∗∗*^	0.06	0.47^*∗∗∗*^	0.38^*∗∗∗*^	0.25^*∗∗∗*^
*HI*	−0.01	−0.14	0.19	0.11	0.07	0.02	−0.09	0.11	0.22^*∗∗*^	0.25^*∗∗∗*^	0.09

*Variable*	*NSPP*	*NSPPo*	*DTF*	*DTM*	*SFP*	*SPE*	*TSW*	*EGR*	*BmWPP*	*SYPP*	*HI*
*LL*	−0.29	−0.62^*∗∗∗*^	−0.73^*∗∗∗*^	−0.70^*∗∗∗*^	−0.53^*∗∗∗*^	0.09	0.17	0.28	0.52^*∗∗*^	0.49^*∗∗*^	−0.08
*LW*	−0.03	−0.33	−0.43^*∗*^	−0.39^*∗*^	−0.37	0.47^*∗∗*^	0.12	0.22	0.66^*∗∗∗*^	0.44^*∗*^	−0.28
*LA*	0.16	0.16	−0.05	0.09	0.21	0.49^*∗∗*^	0.39^*∗*^	0.05	0.49^*∗∗*^	0.61^*∗∗∗*^	0.24
*LAI*	0.11	0.03	−0.33	−0.09	0.17	0.48^*∗∗*^	0.28	0.19	0.70^*∗∗∗*^	0.75^*∗∗∗*^	0.21
*PoL*	−0.15	−0.29	−0.53^*∗∗∗*^	−0.44^*∗*^	−0.25	0.18	0.48	0.37^*∗*^	0.57^*∗∗∗*^	0.56^*∗∗∗*^	−0.01
*PoW*	0.39	0.44	0.17	0.31	0.41^*∗*^	0.24	0.41^*∗*^	0.15	0.31	0.22	0.06
*IL*	−0.28	−0.75^*∗∗∗*^	−0.81^*∗∗∗*^	−0.75^*∗∗∗*^	−0.61^*∗∗∗*^	0.13	0.02	0.47^*∗∗*^	0.55^*∗∗*^	0.41^*∗*^	−0.23
*PHFPo*	0.22	0.61^*∗∗∗*^	0.59^*∗∗∗*^	0.67^*∗∗∗*^	0.58^*∗∗∗*^	0.29	−0.08	−0.37^*∗*^	0.05	0.09	0.18
*PH*	−0.12	−0.11	−0.36^*∗∗*^	−0.17	−0.04	0.28	0.06	0.03	0.48^*∗*^	0.62^*∗∗∗*^	0.29
*NBPP*	0.32	0.04	−0.07	0.08	0.20	0.21	0.42^*∗*^	0.26	0.33	0.49^*∗∗*^	0.31
*NPPP*	0.64^*∗∗∗*^	−0.27	−0.23	−0.18	−0.14	0.16	0.23	0.70^*∗∗∗*^	0.29	0.32	0.03
*NSPP*	1	0.54^*∗∗*^	0.38^*∗*^	0.51^*∗∗*^	0.49^*∗∗*^	0.27	0.36^*∗*^	0.47^*∗∗*^	0.12	0.27	0.34
*NSPPo*	0.47^*∗∗∗*^	1	0.81^*∗∗∗*^	0.90^*∗∗∗*^	0.79^*∗∗∗*^	0.21	0.21	−0.19	−0.2	−0.03	0.37^*∗*^
*DTF*	0.18^*∗∗*^	0.60^*∗∗∗*^	1	0.89^*∗∗∗*^	0.62^*∗∗∗*^	0.01	−0.04	−0.36^*∗*^	−0.51^*∗*^	−0.38^*∗*^	0.22
*DTM*	0.29^*∗∗∗*^	0.73^*∗∗∗*^	0.74^*∗∗∗*^	1	0.85^*∗∗∗*^	0.25	0.12	−0.26	−0.31	−0.14	0.34
*SFP*	0.25^*∗∗*^	0.56^*∗∗∗*^	0.49^*∗∗∗*^	0.72^*∗∗∗*^	1	0.27	0.20	−0.21	−0.12	0.05	0.35
*SPE*	0.12	0.16^*∗*^	−0.01	0.15^*∗*^	0.03	1	0.13	0.25	0.53^*∗∗*^	0.49^*∗∗*^	0.05
*TSW*	0.23^*∗∗*^	0.19^*∗∗*^	−0.02	0.12	0.18^*∗*^	0.09	1	0.61^*∗∗∗*^	0.18	0.49^*∗∗*^	0.49^*∗∗*^
*EGR*	0.43^*∗∗∗*^	−0.11	−0.33^*∗∗∗*^	−0.22^*∗∗*^	−0.19^*∗∗*^	0.17^*∗*^	0.57^*∗∗∗*^	1	0.35	0.51^*∗∗*^	0.25
*BmWPP*	0.05	−0.11	−0.38^*∗∗∗*^	−0.19^*∗∗*^	−0.05	0.36^*∗∗∗*^	0.15^*∗*^	0.31^*∗∗∗*^	1	0.74^*∗∗∗*^	−0.17
*SYPP*	0.27^*∗∗∗*^	0.05	−0.31^*∗∗∗*^	−0.09	0.01	0.32^*∗∗∗*^	0.43^*∗∗∗*^	0.48^*∗∗∗*^	0.66^*∗∗∗*^	1	0.51^*∗∗*^
*HI*	0.33^*∗∗∗*^	0.33^*∗∗∗*^	0.12	0.21^*∗∗*^	0.17^*∗*^	0.03	0.39^*∗∗∗*^	0.22^*∗∗*^	−0.25^*∗∗∗*^	0.51^*∗∗∗*^	1

Description of the variables is given under Table 2.

**Table 10:** Principal component's analysis (PCA) for the 22 quantitative traits considered.

Variable	PC1	PC2	PC3	PC4	PC5	PC6
*LL*	0.32	−0.06	−0.07	0.13	0.03	−0.09
*LW*	0.27	0.08	−0.14	−0.33	−0.06	−0.36
*LA*	0.16	0.29	−0.22	−0.03	0.03	−0.35
*LAI*	0.22	0.25	−0.20	0.03	0.05	0.19
*PoL*	0.27	0.07	−0.02	0.05	0.43	−0.13
*PoW*	−0.02	0.22	0.12	−0.26	0.54	0.12
*IL*	0.31	−0.13	0.06	−0.05	−0.11	0.21
*PHFPo*	−0.15	0.26	−0.31	−0.05	−0.24	−0.08
*PH*	0.18	0.13	−0.31	0.30	−0.19	0.31
*NBPP*	0.11	0.24	0.02	0.11	−0.29	−0.51
*NPPP*	0.15	0.07	0.44	−0.20	−0.35	−0.01
*NSPP*	−0.06	0.28	0.37	−0.17	−0.21	0.06
*NSPPo*	−0.25	0.29	−0.02	−0.02	0.13	0.07
*DTF*	−0.32	0.15	−0.01	−0.06	−0.04	−0.11
*DTM*	−0.28	0.27	−0.01	−0.05	−0.03	0.04
*SFP*	−0.21	0.30	−0.03	−0.01	0.06	0.12
*SPE*	0.10	0.25	−0.10	−0.33	−0.11	0.25
*TSW*	0.10	0.24	0.29	0.26	0.32	−0.26
*EGR*	0.20	0.10	0.47	0.03	−0.05	0.11
*BmWPP*	0.28	0.16	−0.10	−0.27	0.03	0.20
*SYPP*	0.26	0.27	0.01	0.18	−0.05	0.19
*HI*	−0.03	0.23	0.13	0.58	−0.08	0.09
Eigenvalue	7.03	5.36	2.49	1.58	1.36	1.00
Proportion	0.32	0.24	0.11	0.07	0.06	0.05
Cumulative	0.32	0.56	0.68	0.75	0.81	0.86

Description of the variables is presented under Table 2; PC = principal component.

**Table 11:** Inter- and intra- (diagonal element and bold) cluster distance of the tested varieties.

CLS	1	2	3
1	**2.89**		
2	20.14	**4.28**	
3	49.34	91.27	**4.28**

CLS = cluster.

## Data Availability

The datasets generated during and/or analyzed during the current study are available from the corresponding author on reasonable request. Some are included in the supplementary information files.

## Supplementary Materials

Supplementary Materials Supplementary Table 1. List of the varieties along with their major and minor (sub) clusters.
