# Supplementary material for: Assessment of the Effects of Seed Storage Time on Germination Rate and Performance Evaluation of Ethiopian Faba Bean (Vicia faba L.) Varieties for Yield and Related Traits
Source: ScientificWorldJournal. 2022 May 5;2022:6338939. doi: 10.1155/2022/6338939 (PMC9098359; doi:10.1155/2022/6338939)
Supplement: Supplementary Materials — Supplementary Table 1. List of the varieties along with their major and minor (sub) clusters. [file 6338939.f1.docx]

***Supplementary Table 1*:** List of the varieties along with their major and minor (sub) clusters

| **Cluster** | **Sub-cluster** | **Varieties** | **Cluster** | **Sub-cluster** | **Varieties** |
| --- | --- | --- | --- | --- | --- |
| **I** | i | EHOO126 | **II** | i | Alosha |
|  |  | EH99019-4 |  |  | Moti |
|  |  | EH98106-1 |  |  | Tumsa |
|  |  | EH940050V4 |  |  | Moyben |
|  |  | EH95074-9 |  | ii | Shalo |
|  |  | EH98033-3 |  |  | Degaga |
|  | ii | Tesfa |  |  | Cs20-DK |
|  |  | Messay |  |  | Mosisa |
|  |  | Obse |  | iii | Tosha |
|  |  | Didea |  |  | Gebelcho |
|  | iii | EH99102-4 | **III** | i | Numan |
|  |  | EH99071-2 |  |  | Dosha |
|  |  | NC-58-M |  |  | Gora |
|  |  | Woyu |  |  | Ashebeka |
|  |  | Local check* |  | ii | Hachalu |
|  | | |  |  | Dida1 |
|  |  |  | MP |  | Welki |

*MP=monophyletic*
